# Response differences of HepG2 and Primary Mouse Hepatocytes to morphological changes in electrospun PCL scaffolds

**DOI:** 10.1038/s41598-021-81761-z

**Published:** 2021-02-04

**Authors:** Thomas S. R. Bate, Victoria L. Gadd, Stuart J. Forbes, Anthony Callanan

**Affiliations:** 1 Institute for Bioengineering, School of Engineering, University of Edinburgh, Edinburgh, UK; 2 Scottish Centre for Regenerative Medicine, University of Edinburgh, Edinburgh, UK

**Keywords:** Biomaterials, Biomimetics, Tissue engineering, Biological models

## Abstract

Liver disease cases are rapidly expanding across the globe and the only effective cure for end-stage disease is a transplant. Transplant procedures are costly and current supply of donor livers does not satisfy demand. Potential drug treatments and regenerative therapies that are being developed to tackle these pressing issues require effective in-vitro culture platforms. Electrospun scaffolds provide bio-mimetic structures upon which cells are cultured to regulate function in-vitro. This study aims to shed light on the effects of electrospun PCL morphology on the culture of an immortalised hepatic cell line and mouse primary hepatocytes. Each cell type was cultured on large 4–5 µm fibres and small 1–2 µm fibres with random, aligned and highly porous cryogenically spun configurations. Cell attachment, proliferation, morphology and functional protein and gene expression was analysed. Results show that fibre morphology has a measurable influence on cellular morphology and function, with the alteration of key functional markers such as CYP1A2 expression.

## Introduction

Recent decades have seen alarming rises in the rates of chronic liver diseases and hepatocellular carcinoma^1–3^. The breadth of liver related pathologies is large and the associated epidemiology is highly complex^4,5^. Liver transplantation is currently the only feasible cure to end-stage liver disease, but it is costly and donor supply satisfies only 10% of demand^5^. Several strategies are being employed to try to tackle these issues and the search for novel and effective treatments is underway^6^. Finding therapeutic targets for drug based treatment of pre-cirrhotic liver disease is of high importance to effect an economical solution to this problem^6^. Other research is focused on regenerative therapies through the use of stem cells or macrophages and extra-cellular matrix (ECM) scaffolding technologies to remove or reverse the currently irreversible results of liver cirrhosis^7–11^. These methods imply the utilisation of in-vitro methods which are in need of re-development and re-engineering to match in-vivo biological complexity and deliver effective platforms for expansion of regenerative cells and in-vitro drug testing models^11–13^.

In-vitro culture of hepatocytes has developed from standard culture methods to collagen sandwich cultures, organoid cultures and microfluidic organ-on-a chip cultures^14,15^. While each of these methods carries their limitations, the scope for further options has been expanded by research into novel scaffold technologies. Scaffolds take the form of bio-compatible porous hydrogels or polymer microstructures, and supply cells with a bio-mimetic extracellular structure upon which to attach^16^. Amongst the various available methods, recent research has identified electrospun polymer scaffolds as a potential avenue for the production of effective in-vitro liver tissue models^17–21^.

Electrospinning is a polymer fabrication technique that yields fibres from the nano to micro scale that can mimic the structure of the native ECM^22^. Electrospun bio-compatible polymers have been used in the production of 3D cell cultures for various tissues and for various applications^23–28^. It is a reliable and tailorable technique giving a high degree of freedom in altering the dimensional aspects of the fibrous matrix. Fibre size, morphology and connectivity can be easily manipulated, which has contributed to the method’s popularity among tissue engineering researchers^22,29–31^. There are plenty of examples in literature of these attributes being exploited, such as to produce artificial conduits for nerve grafts and in finding optimal fibre characteristics for in-vitro kidney tissue models^25,32^. The ability to alter the fibre characteristics provides the opportunity to investigate the effects of fibre characteristics on cellular function.

The mechanical properties of cell culture substrates can have a direct influence on cellular behaviour and function through a process termed mechanotransduction^33^. It is known that this influence affects the morphology of adherent cells, the cytokinesis process, cellular migration and differentiation^34^. These effects have been demonstrated through studies focused on stem cells, and are known to translate over to fully differentiated somatic cell types. Microenvironment stiffness has been utilised as a tool for directing differentiation of hepatic stem cells/progenitors within in-vitro hydrogel models^35,36^. It has also been shown that substrate stiffness can be used to regulate the function of primary hepatocytes from rats and humans^37,38^. Thus, consideration of the mechanical properties of 3D scaffold technologies and how this regulates cell function is essential to the design of in-vitro systems.

Herein, we report the in-vitro effects of electrospun PCL fibre morphology on the hepg2 cell line and isolated primary mouse hepatocytes (MPH). We have constructed six groups of scaffolds with different morphologies and fibre sizes by altering electrospinning parameters. The morphological categories include random fibres, aligned fibres and higher porosity cryogenic fibres. Each morphological category has been produced with a small fibre size (1–2 µm) and a large fibre size (4–5 µm). This provides an array of different mechanical microenvironments which allows us to understand the extent to which electrospun fibre mechanics influences hepatic cell cultures and apply this information to the design of in-vitro hepatocyte culture systems.

## Results

### Scaffold properties

**Figure 1:**
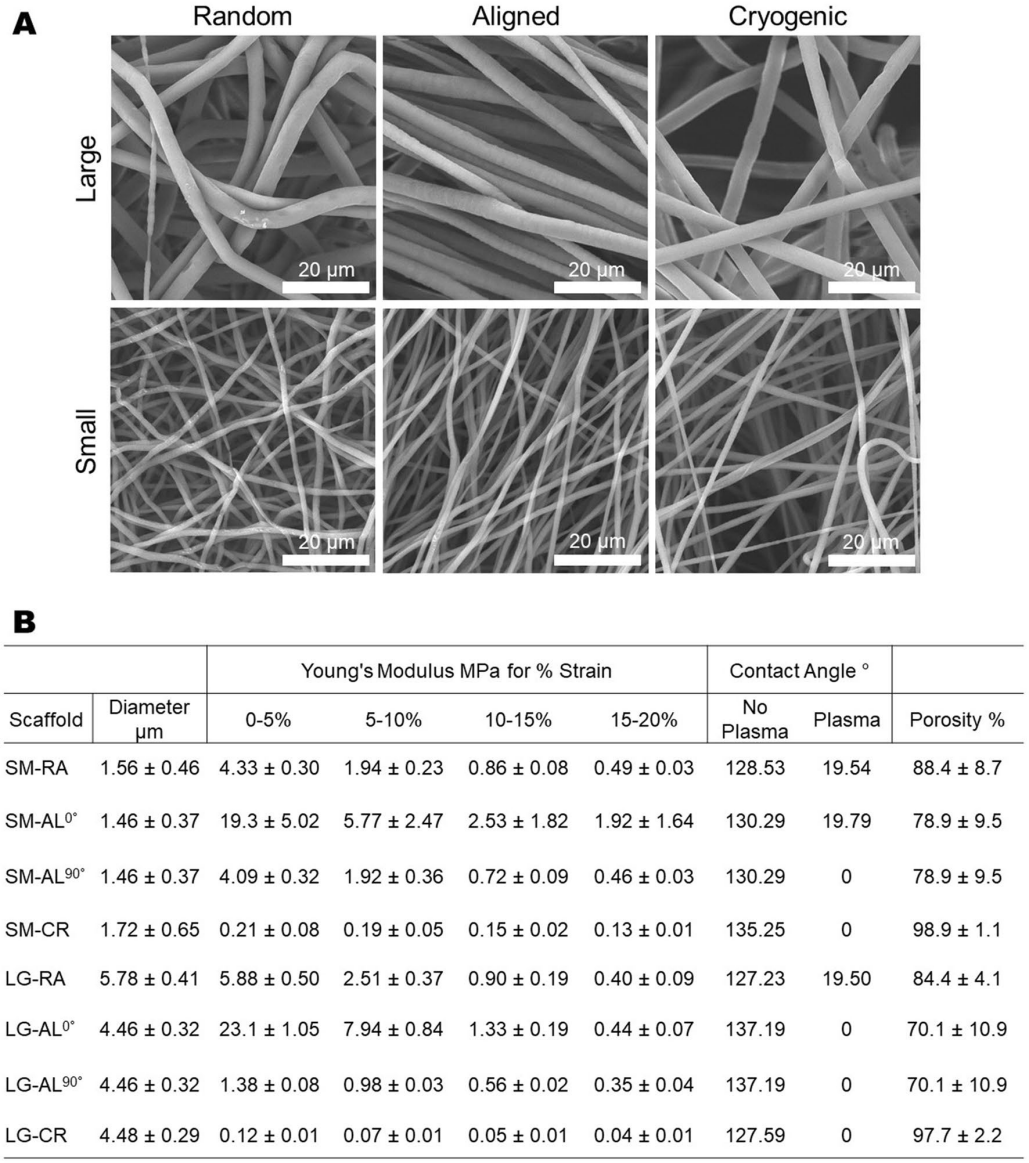
(**A**) SEM images of scaffold fibres for each group. (**B**) Fibre diameter measurements from Diameter J plugin, Tensile mechanical stiffness for each scaffold group (N = 5 presented as mean ± SD), Contact angle measurements for each scaffold group (0 indicates droplet completely absorbed) before and after plasma treatment and porosity measurements.

The scaffold fabrication methods used in this study produced three distinct PCL scaffold morphologies, as shown in Fig. 1A, with large (4–5 µm) and small (1–2 µm) fibres. Control of fibre size was implemented through alteration of polymer concentration, solvent choice and electrospinning parameters. The morphology of the deposited fibres was controlled by varying mandrel speed and temperature. Figure 1B shows the effect that fibre size and morphology have on the mechanical properties of the resulting scaffolds. Morphological characteristics have a stronger effect on the mechanical properties, however it is clear that fibre size also has influence.

### Cell morphology

**Figure 2:**
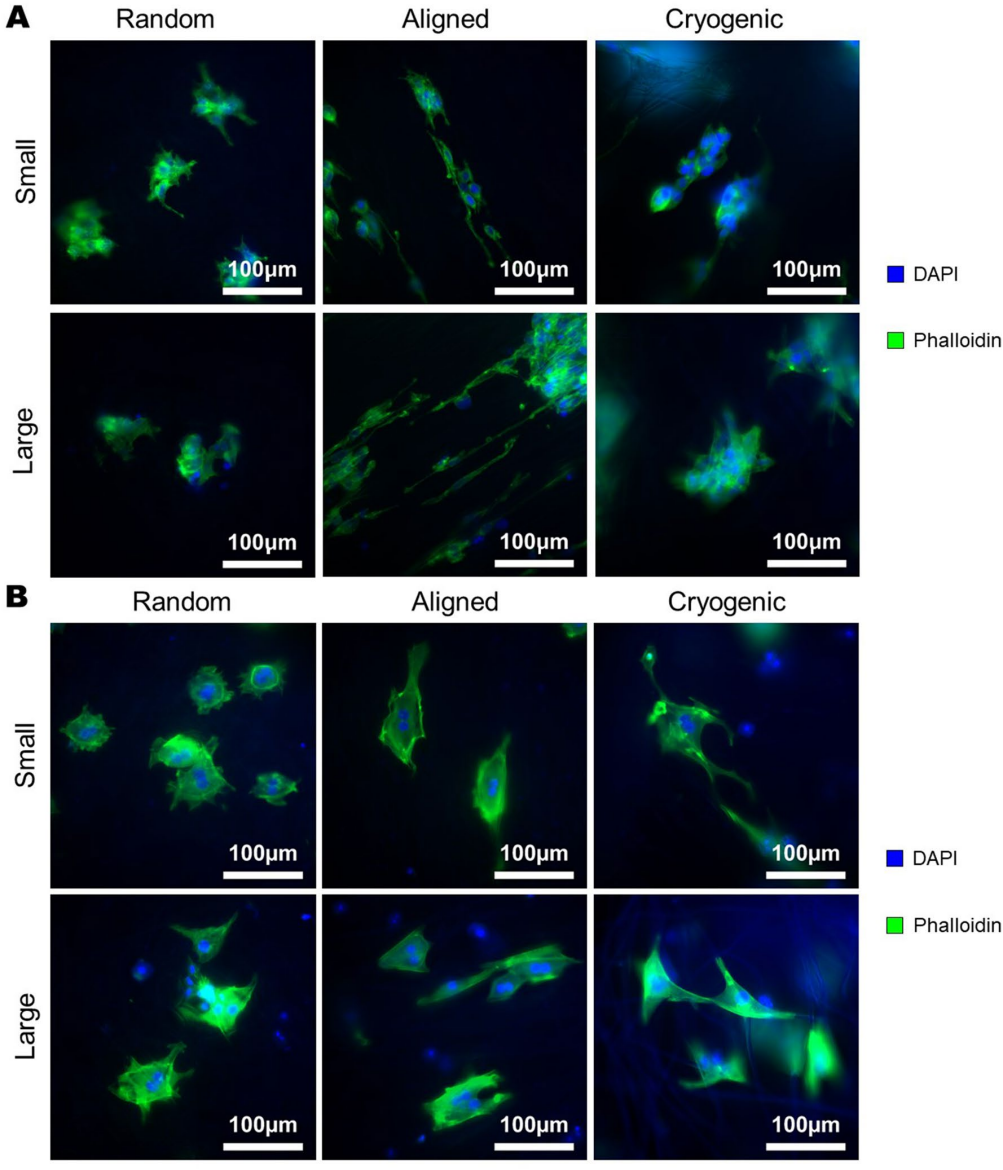
DAPI and Phalloidin fluorescence images showing cellular morphology upon scaffolds at 48 h (**A**) Hepg2, (**B**) MPHs. Images taken with ×40 magnification objective.

The morphological effects of each scaffold on hepg2s and MPHs is displayed in Fig. 2. Cells adopt a more rounded morphology on the random scaffolds and are significantly elongated on the aligned fibres. The morphology of cells that have attached to fibres in the cryogenic scaffolds adheres to the morphology of the fibre. However, these cryogenic scaffolds also show indications of cellular infiltration and trapping of unattached cells within the large pores.

### Cell viability

**Figure 3:**
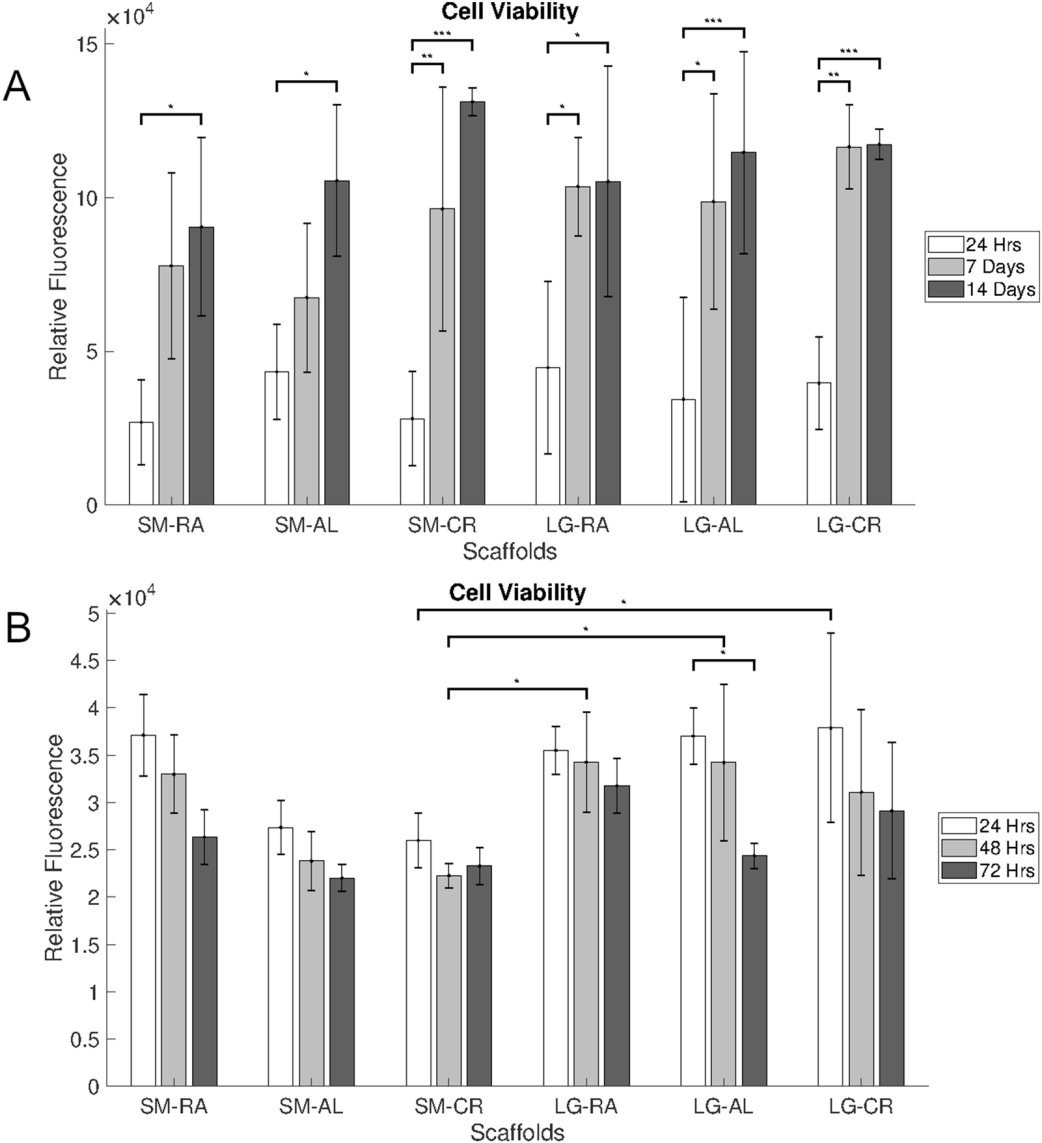
Cell titre blue fluorescence results for (**A**) hepg2 over 14 days and (**B**) MPHs over 3 days. N = 5, Error bars = SD and *p < 0.05, **p < 0.01 and ***p < 0.001.

Cell titre blue results in Fig. 3 showed hepg2 cells exhibited increased cell viability over the 14 day culture period. Due to the highly proliferative nature of the hepg2s this is likely due to increases in the cell number upon the scaffold. Larger fibres tended to increase level of viability measured, however no statistically significant differences were observed between the groups.

In contrast to the hepg2s the MPHs exhibited a steady decline in cell viability over a 3 day culture period. This is largely due to cell death within the culture of MPHs, which is common amongst primary hepatocyte cultures^39^. Again, larger fibres tended to increase the viability levels with the exception of the SM-RA group. Statistically significant differences were observed between the SM-CR scaffold and each of the LG-RA and LG-AL scaffold groups at 48 h (p < 0.05). Interestingly, significantly increased fluorescence was observed on the LG-CR scaffold in comparison to the SM-CR scaffold (p < 0.05), which suggests higher attachment on the larger fibres.

### DNA

**Figure 4:**
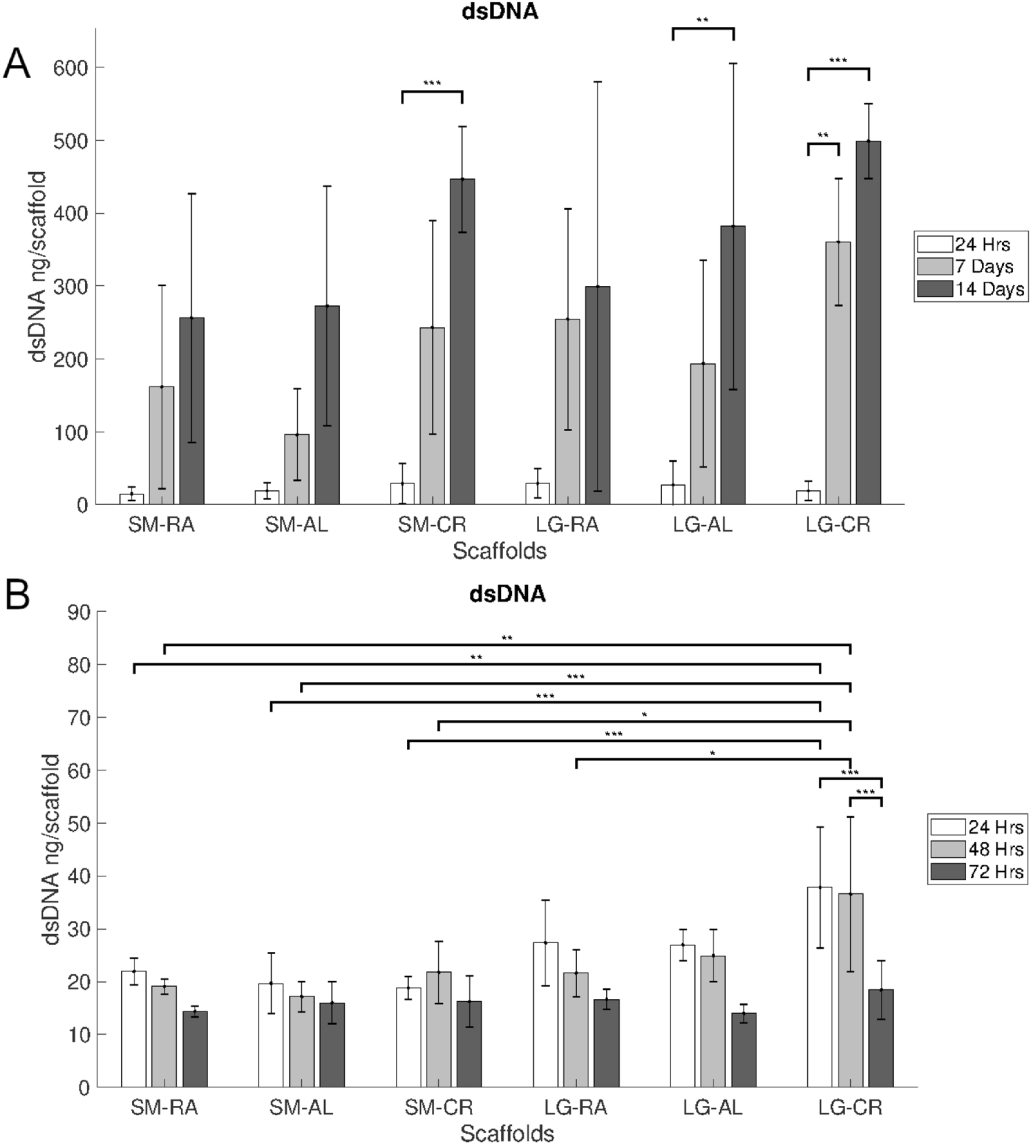
Picogreen dsDNA measurements for (**A**) hepg2 over 14 days and (**B**) MPHs over 3 days. N = 5, Error bars = SD and *p < 0.05, **p < 0.01 and ***p < 0.001.

dsDNA levels have been quantified in order to understand cell attachment and proliferation behaviour. This can also confirm if cell viability changes are characterised by changes in cell number or cell metabolism. Measured dsDNA levels in the hepg2 cultures (Fig. 4A) showed consistent increases across the 14 day culture period. Cryogenic scaffold groups showed higher rates of increase with statistically significant increases from 24-h to 14-days (p < 0.001).

The capacity for MPHs attachment was similar between scaffolds as evidenced by consistent levels of dsDNA at 24 h (Fig. 4B). In contrast to HepG2, MPHs exhibited consistent decreases in the dsDNA levels over the 3 day culture period, displayed in (Fig. 4B). The LG-CR scaffolds held significantly higher levels of dsDNA at 24 and 48 h compared with each of the SM scaffold groups (p < 0.05–0.001) and the LG-RA group (p < 0.05).

### Albumin

**Figure 5:**
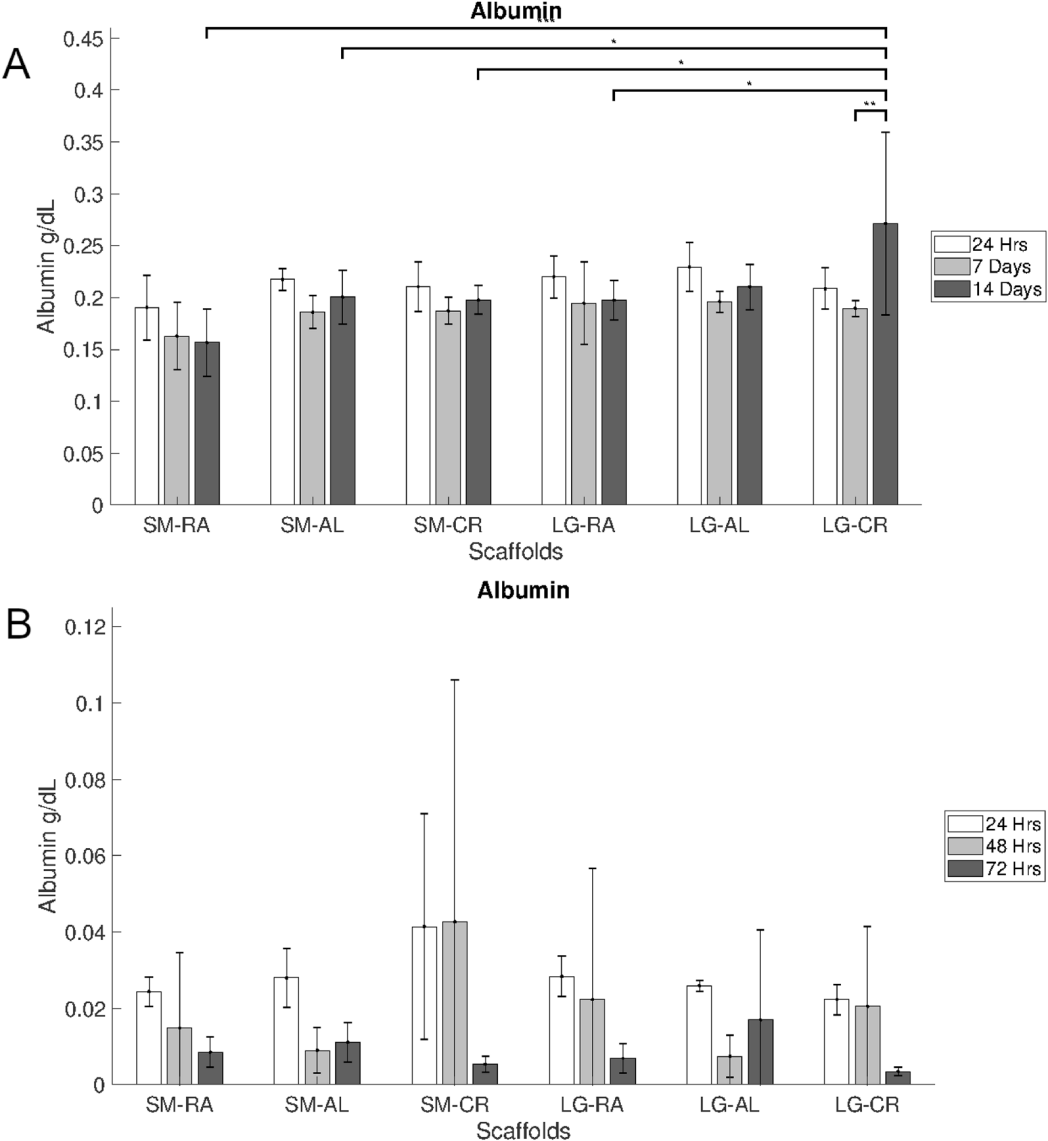
Bromocresol green 24 h albumin secretion measurements for (**A**) hepg2 over 14 days (N = 5) and (**B**) MPHs over 3 days (N = 3). Error bars = SD and *p < 0.05, **p < 0.01 and ***p < 0.001.

Albumin secretion displayed in Fig. 5A, measured by bromocresol green assay, showed hepg2 secreted between 0.2 and 0.3 g/dL over a 24 h period at all timepoints. At 14 days hepg2s on the LG-CR scaffold showed significantly increased secretion over 24 h compared with all other groups except LG-AL (p < 0.05–0.001).

Figure 5B shows that MPHs exhibited 0.02–0.05 g/dL albumin secretion over a 24 h period for all timepoints. No significant differences in albumin secretion were observed between groups.

### Gene expression

**Figure 6:**
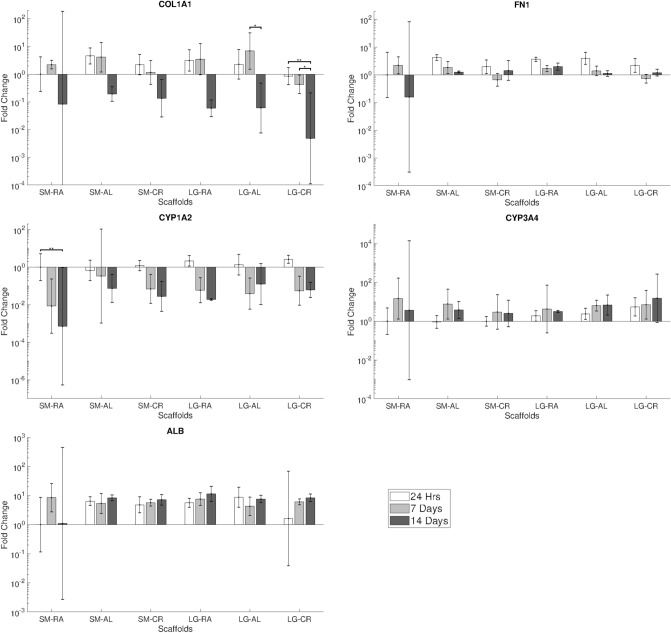
RT-qPCR gene expression results showing fold change of COL1A1, FN1, CYP1A2, CYP3A4 and ALB for hepg2 over 14 days. Results normalised to hepg2 on SM.RA at 24 h. N ≤ 5, Error bars = SD and *p < 0.05, **p < 0.01 and ***p < 0.001.

Gene expression analysis for hepg2 cells shown in Fig. 6 confirms the expression of COL1A1, FN1, ALB, CYP3A4 and CYP1A2. Relative expression of genes between groups showed no significant differences. CYP3A4 and ALB exhibited increases in expression over the 14 day culture period, whilst decreases were observed in COL1A1, FN1 and CYP1A2.

**Figure 7:**
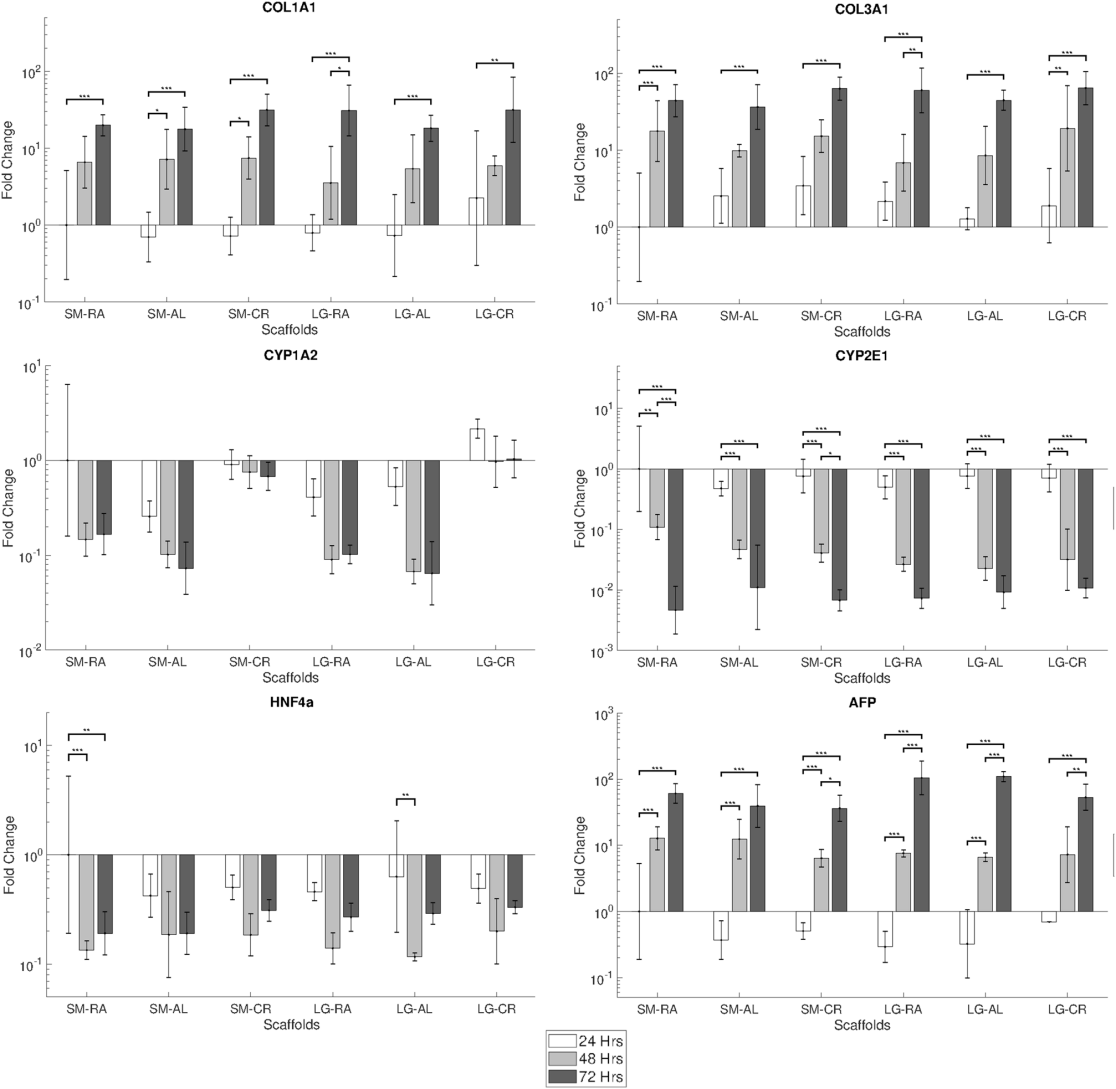
RT-qPCR gene expression results showing fold change of COL1A1, COL3A1, AFP1, HNF4a, CYP1A2 and CYP2E1 for MPHs over 3 days. Results normalised to MPHs on SM.RA at 24 h. N ≤ 5, Error bars = SD and *p < 0.05, **p < 0.01 and ***p < 0.001.

MPHs were confirmed to show expression of COL1A1, COL3A1, AFP1, HNF4a, CYP1A2 and CYP2E1, seen in Fig. 7. COL1A1, COL3A1 and AFP1 were seen to increase over the 3 day culture period, and HNF4a, CYP1A2 and CYP2E1 were seen to decrease. Significant increases in the expression of CYP1A2 between the cryogenic groups and all of the other groups were observed after 48 h of culture time (p < 0.05–0.001, these are provided in the Supplementary Information).

## Discussion

The mechanical properties of the scaffolds are a result of the combination of fibre bending stiffness and the mode of fibre connectivity within the fibrous matrix^40^. Depending on these two parameters, under load each scaffold will exhibit either bending dominant or stretching dominant deformation which has a large impact on the macro-mechanical behaviour of the fibrous material^41^. Apart from mechanical considerations, the morphological changes between each scaffold type can be observed at the cell scale. Therefore, it was reasonable to assume that these structural characteristics would have an influence on the morphology of attached cells. The scaffolds produced thus displayed properties that would make them a useful platform on which to test the mechanical and morphological influence of electrospun fibres on hepatocyte cultures. This is important in order to develop in-vitro platforms that can induce mechanically and morphologically relevant cultures, and produce effective culture environments for cell therapy and drug testing workflows. The analysis was conducted such that standard tissue culture plastic (TCP) controls were omitted in order the focus the analysis on the morphological factors, previous reports in literature have explored comparisons between 3D scaffold cultures and 2D TCP culture^21,42,43^.

Cellular attachment of hepg2 was observed on all scaffolds and each scaffold is shown to have facilitated an equal measure of attachment, as shown by levels of DNA at 24 h in Fig. 4. The hepg2 were then allowed to proliferate over 14 days to understand if the electrospun scaffolds exert influence across the short term and the long term. No significant differences were exhibited between the scaffold groups throughout the entire culture period. Although trends in the DNA and viability results indicate that larger pores in the scaffolds promote higher rates of proliferative activity. It is widely understood that scaffold porosity and pore size can act on the migration and proliferation behaviours of cells within scaffolds^44,45^.

MPHs were seen also to attach PCL fibres successfully and were observed upon the scaffolds for three days. DNA results shown in Fig. 4B indicate significantly higher levels of cellular attachment in the LG-CR group, this is also reflected in the overall improved viability on large scaffolds seen in Fig. 3B. It is thought this is likely due to more cells becoming trapped in the larger pores of the scaffold. Over the course of three days the MPHs were seen to decline in population on the scaffolds, it was expected that this was to happen with MPHs, which are notoriously non-proliferative in-vitro and rapidly progress to apoptosis^39^*.* The SM-CR group showed reduced levels of viability compared to the LG-RA and LG-AL groups at 48 h. As this is not reflected in the levels of DNA it is possible that the smaller fibres and reduced connectivity of the SM-CR scaffold has an inhibitory effect on metabolic activity, however more rigorous analysis of metabolic activity would be necessary to confirm such effects.

Albumin secretion was confirmed for both cell types upon the electrospun PCL scaffolds, seen in Fig. 5. Albumin is a physiologically important molecule secreted by hepatocytes which regulates colloidal osmotic pressure and the transportation of molecules throughout the body, amongst other important physiological processes^46,47^. The production of albumin in-vitro is thus essential for an effective model of hepatocyte function. No major differences in albumin secretion were observed between groups, apart from the hepg2 on the LG-CR scaffold at 14 days. This is not supported by evidence of significantly increased cell population, therefore scaffold porosity may drive albumin secretion through more extensive 3D cell agglomeration, which has been shown to augment albumin production in hepatic cell lines^48^.

Gene expression was analysed for Albumin, CYP1A2 and CYP3A4 genes to show expression of hepatic functional markers for hepg2. Expression of COL1A1 and FN1 were also analysed to observe the effects on ECM production. Scaffold morphology was not highly influential on the expression of genes analysed, as confirmed in Fig. 6 Hepg2 cells showed fairly consistent expression profiles for all genes between scaffold groups. There were trends noted in the expression of COL1A1 where expression after 14 days was seen to be reduced with fibre size and particularly on the LG-CR group.

In accordance with hepg2, MPHs also exhibited a largely unaffected expression of functional markers (AFP, HNF4a and CYP2E1) and ECM genes (COL1A1 and COL3A1) across the morphological categories. This is with the exception of CYP1A2, in which statistically a significant increase in expression was observed on the cryogenic scaffolds SM-CR and LG-CR after 48 h of culture, which can be seen in Fig. 7. CYP1A2 is a cytochrome P450 protein primarily associated with the metabolism of caffeine, drugs and other xenobiotics^49–51^. The cryogenic scaffolds share higher porosity in comparison to the other morphological categories shown by the porosity data in Fig. 1. A higher porosity allows for deeper scaffold infiltration and facilitates a higher degree of 3-D agglomeration of the cells within the scaffold, constructing a more organoidal arrangement. CYP1A2 expression and activity has been found to be increased in 3D culture conditions in comparison to 2D methods^43,52^. Accordingly, it is proposed that the 3D structural arrangements driven by the higher porosity in cryogenic electrospun scaffolds may influence CYP1A2 expression. This supports evidence in literature that suggests scaffold pore size and geometry can have an impact on several aspects of hepatocyte function^53,54^. It should be noted that the gene expression panels in Figs. 6 and 7 are not for the same genes due to the known differences in gene expression profiles between hepG2 and primary hepatocytes^55–58^. A comparison between the two cell types would not fall within the scope of this study.

Electrospun PCL scaffold morphology has a direct impact on the morphology of hepg2 and MPH cell cultures which, in turn, show to produce significant biological responses. This is confirmed by results showing altered scaffold-cell attachment, proliferation rates and changes to the expression of major hepatocyte functional markers, such as CYP1A2. The results imply that fibre size and porosity both have measurable influence on the cultures. In particular, the higher occurrences of 3-dimensional cell–cell interactions in scaffolds with higher porosity show to consistently affect cell function in a significant manner. This study opens questions about how electrospun scaffold morphology should be utilised to harness bio-realistic in-vitro cultures of hepatocellular-carcinoma and primary mouse hepatocytes.

## Method

### Electrospinning

**Figure 8:**
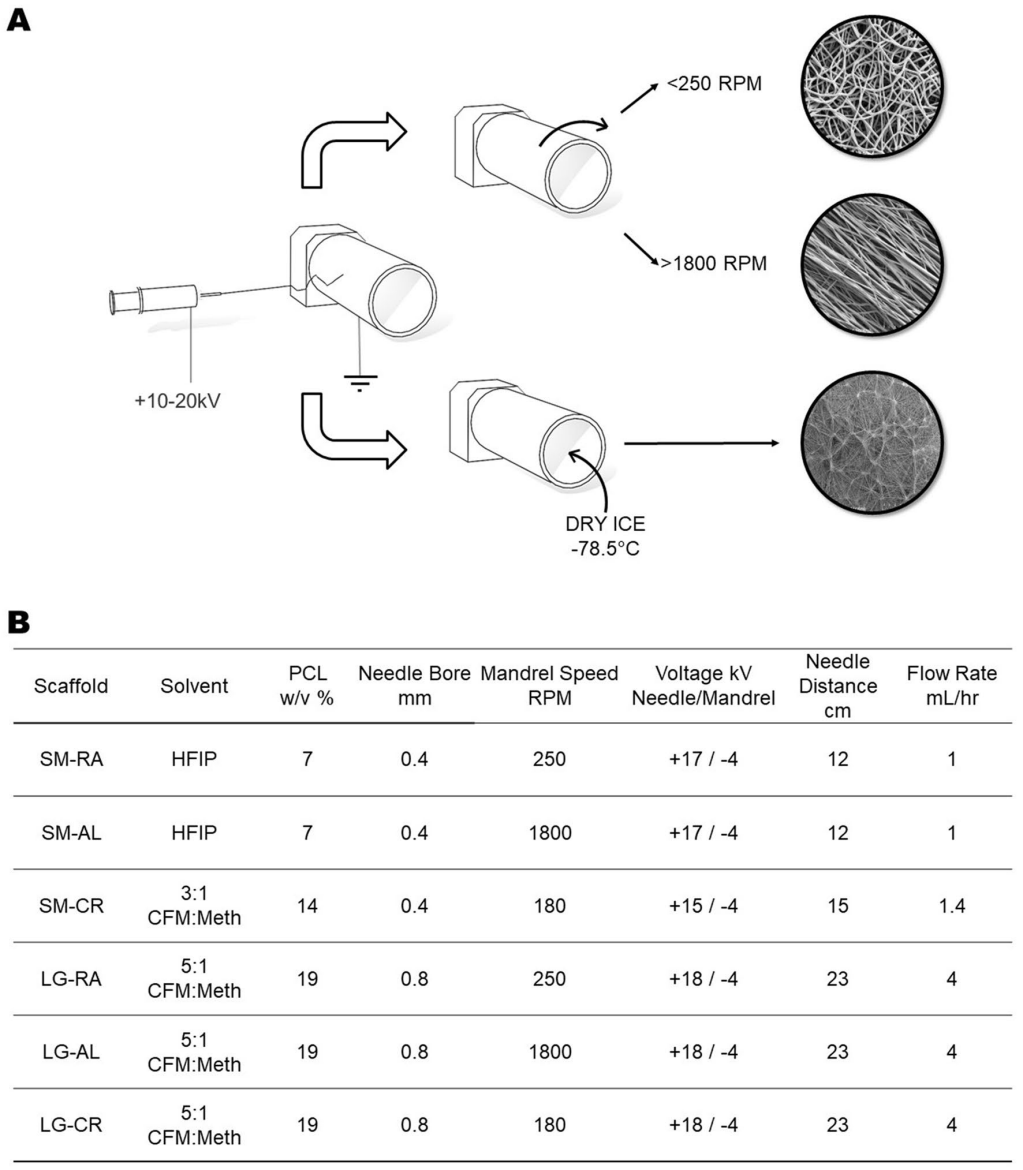
(**A**) Diagram depicting the electrospinning process for random, aligned and cryogenic fibres (top to bottom, respectively). (**B**) Table showing electrospinning parameters for each scaffold group. (*CFM:Meth *Cloroform:Methanol).

Electrospun PCL scaffolds of differing morphology were produced using a previously described methods^23,59^. The scaffolds were fabricated by solution electrospinning on an IME Technologies electrospinning apparatus with a rotating mandrel attachment. To produce the six different architectures a variety of electrospinning parameters were used shown in Fig. 8. PCL was dissolved in different solvent systems Chloroform:Methanol 5:1, Chloroform:Methanol 3:1 and Hexafluoroisopropanol (HFIP) to achieve different fibre sizes, an approach demonstrated in literature^23,54,60^. For each scaffold, 4 mL of solution was used to spin a 100 mm wide sheet of electrospun material from which 10 mm discs were punched out. These discs were stored at room temperature awaiting plasma treatment and seeding.

### Scanning electron microscopy

Samples were sputter coated with gold in an EmScope 5000 sputter coater for 30 s before imaging. Post sputter coating, SEM images of the samples were taken at 15 kV accelerating voltage using a Hitachi TM4000 Plus benchtop scanning electron microscope.

### Fibre diameter

Fibre diameter was measured on ImageJ software using the DiameterJ plugin^61^. SEM images were segmented and analysed to find the average fibre diameter for each scaffold.

### Porosity measurement

50 × 10 mm (N = 3) sections of scaffold material were cut using a scalpel. The samples were measured for thickness using a DMK 41AU02 (ImagingSource) camera and weighed. With knowledge of the scaffold weight and dimensions and the density of the PCL (Sigma) the porosity was calculated using the following equation:

$$p = \left( {1 - \frac{{V_{PCL} }}{{V_{Scaffold} }}} \right) \times 100,$$

where *p* is porosity, V_PCL_ the volume of PCL calculated from the weight and density (1.145 g/mL) and V_Scaffold_ the volume of the scaffold.

### Mechanical testing

Electrospun material was subject to tensile testing using the Instron 3367 universal testing system. Briefly, 10 × 50 mm samples were cut using a scalpel and mounted 10 mm at each end in pneumatic clamps giving a gauge length of 30 mm. The samples (N = 5) were then elongated parallel to the mandrel rotation direction at 50% strain per minute (15 mm/m), strain was measured as longitudinal elongation as a proportion of gauge length. For the aligned fibres the tensile test was conducted both parallel and perpendicular to the mandrel rotation direction to capture the anisotropic properties of the fibres. Sample load was measured using a 50 N rated Instron 2530-50N load cell. Sample thickness was measured using a DMK 41AU02 (ImagingSource) camera. The camera was mounted perpendicular to the scaffold and pictures taken with a scale. Thickness measurements were then taken digitally using ImageJ software. After data collection Youngs’ Moduli for each scaffold were calculated on MATLAB for different strain ranges by taking a regression of the stress–strain data between 0–5%, 5–10%, 10–15% and 15–20% strain.

### Contact angle measurement

5 µL of dH_2_O were pipetted on to the surface of each scaffold and images were taken at 5 Hz using a DMK 41AU02 (ImagingSource) camera. Contact angles for both non-plasma treated scaffolds and plasma treated scaffolds were subsequently measured using LBADSA plugin with ImageJ^62^.

### Plasma treatment

To improve the hydrophilicity of the PCL surface, the scaffolds were oxygen plasma treated (Harrick Plasma) prior to seeding. Briefly, the scaffolds were sterilised in 70% Ethanol for 20 min before being freeze dried for 24 h. The sterile scaffolds were then exposed to oxygen plasma at 500 mTorr at medium RF power (20 W) for 30 s. Once plasma treated the scaffolds were immediately placed into 1% Anti-Anti solution in PBS, preventing hydrophobic recovery.

### Immortalised cell line culture: HepG2

1.0 × 10^6^ hepG2 cells (Sigma) were raised at P6 in 3 T75 tissue culture flasks until 80% confluency in complete media, containing EMEM (Gibco) supplemented with 10% Fetal Bovine Serum (GE healthcare), 5% Non-essential amino acids (Gibco), 5% l-Glutamine (Gibco) and 5 mL 10,000 U/mL Penicillin–Streptomycin (Gibco). Cells were then trypsinised using standard methods and counted with a haemocytometer. The cells were re-suspended in complete media and seeded onto the scaffolds at a concentration of 1 × 10^5^ cells/scaffold. The seeded cells were incubated for 2.5 h at 37 °C and 5% CO_2_ to allow for cell attachment. 300 µL complete media was subsequently added and the cells incubated at 37 °C and 5% CO_2_ for 14 days, changing media every 2–3 days.

### Primary Mouse Hepatocyte isolation and culture

Primary Mouse Hepatocytes were isolated following the standard two-step collagenase perfusion technique^63^. Briefly, mice received a terminal dose of anaesthetic and underwent laparotomy. The portal vein was cannulated and then perfused with 50 mL Liver Perfusion Medium (Gibco) followed by 50 mL Liver Digest Medium (Gibco) pre-warmed to 37 °C. The liver was removed and then hepatocytes were mechanically disassociated and released from the Glisson capsule, filtered through 70 μM filter and purified with a density gradient centrifugation. The cells were seeded at a concentration of 5 × 10^4^ cells/scaffold.

All experiments with animals were approved by the University of Edinburgh Animal Welfare and Ethical Review Body and the UK Home Office. All experiments with animals took place at the Centre for Regenerative Medicine, Edinburgh and were performed according to procedural guidelines and severity protocols from the UK Home Office Animals Scientific Procedures Act.

### Cell viability

Cell viability was assessed using the Cell Titre Blue assay (Promega). The assay was conducted according to the manufacturer’s instructions. Briefly, cell laden scaffolds were incubated at 37 °C and 5% CO_2_ in a 5:1 ratio of complete media and Cell Titre Blue reagent for 4 h. 100 µL of the resulting media solution was then measured for fluorescence at ex. 525 nm em. 580–640 nm on a Modulus II Microplate reader.

### DNA quantitation

The amount of dsDNA present on each scaffold was measured using the Quant-IT Picogreen assay (Thermo-Fischer). Scaffolds were washed three times in PBS before being lyophilised. The lyophilised scaffolds were then placed in a Papain digest solution consisting of 2.5 U/mL Papain, 5 mM Cysteine HCl, 100 mM EDTA and 100 mM Sodium Acetate. Scaffolds were incubated at 65 °C in 1 mL of Papain digest solution for 24 h. Samples were then combined with Picogreen reagent as per manufacturer’s instructions. Sample fluorescence was read at ex 490 nm em 510–570 nm on a Modulus II Microplate reader.

### Albumin quantitation

Albumin secretion over 24 h was quantified using the Bromocresol Green (BCG) reagent (Sigma) Media samples were removed from the scaffolds and stored at − 80 °C before measurement. Samples were spun down at 200*g* for 1 min and 5 µL added to the BCG reagent. Absorbance was measured at 620 nm and normalised to a standard curve.

### Cell imaging

Samples were washed 3 times in PBS before fixation in 10% formalin (Sigma) for 15 min, after fixation scaffolds were washed 3 times in PBS and stored at 4 °C before staining. Samples were stained with 300 nM 4′,6-diamidino-2-phenylindole (DAPI, Thermo-Fischer) in PBS and Phalloidin 514 nm conjugate (Sigma) in 1% BSA PBS solution for nucleic and f-actin visualisation respectively. Briefly, samples were washed 3 times in PBS, exposed to DAPI solution for 10 min, washed 3 times in PBS, exposed to Phalloidin solution for 30 min and finally washed 3 times in PBS. Samples were imaged on a Zeiss AxxioImager epifluorescence microscope with 40× objective.

### Gene expression

Samples were placed directly into 1 mL Tri-reagent and 200 µL of chloroform added, samples were spun down at 10,000*g* for 15 min and the mRNA containing supernatant was removed and placed into 400 µL of 70% ethanol. The ethanol and supernatant mRNA mixture was subsequently purified using Qiagen RNEasy Spin columns following the manufacturer’s instructions. After purification, 100 ng of mRNA was translated to cDNA using the Improm II Reverse Transcription kit (Promega) as per the manufacturer’s instructions. qPCR was then carried out with SYBR Green (Qiagen) using a Lightcycler 480 (Roche). Primers for hepG2 were designed in a previous study and sequences are detailed in Supplementary Information. For the MPHs primers are listed in the Supplementary Information. Controls for gene expression analysis were the respective cells cultured on tissue culture plastic over 24 h. Results are expressed as fold-change values derived through the 2^−∆∆Ct^ method.

### Statistical analysis

All numerical data were subjected to one-way ANOVA and Tukey’s HSD post hoc testing. Statistical differences are displayed as: *p < 0.05, **p < 0.01 and ***p < 0.001.

## Supplementary Information

Supplementary Information.
