# Supplementary material for: Response differences of HepG2 and Primary Mouse Hepatocytes to morphological changes in electrospun PCL scaffolds
Source: Sci Rep. 2021 Feb 4;11:3059. doi: 10.1038/s41598-021-81761-z (PMC7862353; doi:10.1038/s41598-021-81761-z)
Supplement: Supplementary file 1 — Supplementary Information. [file 41598_2021_81761_MOESM1_ESM.docx]

Supplementary Information

Response differences of HepG2 and Primary Mouse Hepatocytes to morphological changes in electrospun PCL scaffolds

Thomas SR Bate^1^, Victoria L Gadd^2^, Stuart J Forbes^2^, Anthony Callanan^1^

^1^ Institute for Bioengineering, School of Engineering, University of Edinburgh

^2^ Scottish Centre for Regenerative Medicine, University of Edinburgh

Corresponding Author E-mail ID: [Anthony.Callanan@ed.ac.uk](mailto:Anthony.Callanan@ed.ac.uk)

|  | 24 HRS - P VALUES | | | | | |
| --- | --- | --- | --- | --- | --- | --- |
|  | SMRA | SMAL | SMCR | LGRA | LGAL | LGCR |
| SMRA |  | 0.069437 | 1 | 0.762641 | 0.970236 | 0.874283 |
| SMAL |  |  | 0.132764 | 0.999565 | 0.919029 | 8.97E-05 |
| SMCR |  |  |  | 0.889078 | 0.994607 | 0.731439 |
| LGRA |  |  |  |  | 1 | 0.014851 |
| LGAL |  |  |  |  |  | 0.050909 |
| LGCR |  |  |  |  |  |  |

|  | 48 HRS - P VALUES | | | | | |
| --- | --- | --- | --- | --- | --- | --- |
|  | SMRA | SMAL | SMCR | LGRA | LGAL | LGCR |
| SMRA |  | 0.999958 | 0.008462 | 0.998271 | 0.857776 | 0.000909 |
| SMAL |  |  | 0.0003 | 1 | 0.999802 | 2.51E-05 |
| SMCR |  |  |  | 9.09E-05 | 5.30E-06 | 1 |
| LGRA |  |  |  |  | 0.999999 | 7.61E-06 |
| LGAL |  |  |  |  |  | 1.02E-06 |
| LGCR |  |  |  |  |  |  |

|  | 72 HRS - P VALUES | | | | | |
| --- | --- | --- | --- | --- | --- | --- |
|  | SMRA | SMAL | SMCR | LGRA | LGAL | LGCR |
| SMRA |  | 0.793185 | 0.050909 | 0.998329 | 0.583004 | 0.001638 |
| SMAL |  |  | 3.14E-05 | 0.999987 | 1 | 1.08E-06 |
| SMCR |  |  |  | 0.000852 | 9.41E-06 | 0.999764 |
| LGRA |  |  |  |  | 0.999178 | 1.43E-05 |
| LGAL |  |  |  |  |  | 8.01E-07 |
| LGCR |  |  |  |  |  |  |

*Table S.1: ANOVA p-values for Figure.7 CYP1A2 comparisons.*

| GAPDH | QT00199388 |
| --- | --- |
| AFP-1 | QT00174020 |
| HNF4A | QT00144739 |
| CYP1A2 | QT00100674 |
| CYP2E1 | QT00112539 |
| COL1A1 | QT02589482 |
| COL3A1 | QT02331301 |

*Table S.2: Primer codes for Mouse Primary Hepatocyte qPCR primers, supplied by QIAGEN*

| GAPDH | For – GTCTCCTCTGACTTCAACAG, Rev - GTTGTCATACCAGGAAATGAG |
| --- | --- |
| ALBUMIN | For - CCTGTTGCCAAAGCTCGATG, Rev - GAAATCTCTGGCTCAGGCGA |
| CYP1A2 | For - CTTCGCTACCTGCCTAACCC, Rev - GTCCCGGACACTGTTCTTGT |
| CYP3A4 | For- TTTTTGGATCCATTCTTTCTCTCAA, Rev- TCCACTCGGTGCTTTTGTGT |
| FN1 | For - GGACACAGAGGTTTCAGTGGT, Rev - GCACCATCATTTCCACGAGC |
| COL1A1 | For - GAACAAACACTAATGTTAATTGCCC, Rev - TCTTGGCAGAGAGACATGCTT |

*Table S.3: Primer sequences for HepG2 qPCR primers, supplied by Sigma*
